# Does it blend? Exploring therapist fidelity in blended CBT for anxiety disorders

**DOI:** 10.1016/j.invent.2021.100418

**Published:** 2021-06-26

**Authors:** Geke Romijn, Simon Provoost, Neeltje Batelaan, Jeroen Koning, Anton van Balkom, Heleen Riper

**Affiliations:** a Clinical Psychology Section, Department of Clinical, Neuro- and Developmental Psychology, Vrije Universiteit, Amsterdam, the Netherlands; b Specialised Mental Health Institution, GGz Breburg, Tilburg, the Netherlands; c Amsterdam Public Health Research Institute, Amsterdam University Medical Center, VU University Medical Center, Amsterdam, the Netherlands; d Department of Research and Innovation, GGZ inGeest, Amsterdam, the Netherlands; e Department of Psychiatry, Amsterdam University Medical Center, Amsterdam, the Netherlands; f Institute for Psychiatry, Vincent van Gogh, Venray, the Netherlands

**Keywords:** Internet-based treatment, Blended treatment, Cognitive behavioural therapy, Anxiety disorders, Routine care, Fidelity

## Abstract

Blended cognitive-behavioural therapy (bCBT) combines face-to-face CBT (FtFCBT) and Internet-based CBT (iCBT) into one integrated treatment protocol, opening up new ways to deliver therapy, increase cost-effectiveness and resolve scarcity of therapist availability. When traditional therapy is transformed into a new format, there is a need to evaluate whether principles of the new protocol are consistently applied.

This study aimed to explore therapist fidelity to bCBT protocols for anxiety disorders in specialised mental health care and to assess whether fidelity is related to patient characteristics. Adult patients (*N* = 44) received bCBT within a randomised controlled trial. Ratio of FtF to online sessions, session frequency and therapist adherence to instructions were assessed.

Overall therapist fidelity with regard to ratio of blending, session frequency and instructions was high. Correlations were found between patients' share of online sessions and both session frequency (*r* = 0.373, *p* = .013), as well as patient computer experience (*r* = 0.314, *p* = .038). Adherence to instructions in FtF sessions was based on a subset of patients (*n* = 23) and should therefore be interpreted with caution.

The blended approach was generally delivered as intended, indicating that the format is feasible in specialised mental health.

## Highlights

• Therapist fidelity to blended cognitive behavioural therapy protocols for severe anxiety disorders was high.
• Treatment of higher intensity had a higher share of online sessions.
• Patients with more computer experience were likely to receive higher shares of online sessions.
• Results support feasibility of the blended approach in specialised mental health care.
• Results enhance confidence in earlier findings on effectiveness and cost-effectiveness of blended cognitive behavioural therapy for severe anxiety disorders.

## 1 Introduction

Cognitive-behavioural therapy (CBT) is an effective psychological treatment for anxiety disorders (Stewart and Chambless, 2009). Internet-based CBT (iCBT), where treatment is offered on an online platform, has the potential to maximize cost-effectiveness by reducing the burden of travel and reducing therapist hours. The uptake of iCBT in routine care is low, however, possibly because iCBT is not considered suitable for all patients; for example, providing web-based treatment without face-to-face (FtF) contact may not be deemed acceptable for patients with severe symptoms (Gun et al., 2011). A more recent approach, blended cognitive-behavioural therapy (bCBT), combines face-to-face CBT (FtFCBT) and iCBT, partially replacing FtF sessions with online sessions. Such a treatment format could address the limitations related to iCBT and may also fit better into current routine practice. In a previously conducted randomised controlled trial (RCT) (Romijn et al., in press) we evaluated the acceptability and effectiveness of bCBT (*n* = 52) in comparison with FtFCBT (*n* = 62) for anxiety disorders in specialised mental health care and found promising results. Patients in both groups reported high levels of treatment satisfaction, and both conditions yielded large within group effect sizes at posttest and at one-year follow-up. A small RCT (*N* = 36) of bCBT for panic disorders by another research group achieved results in line with our findings regarding acceptability and effectiveness, with medium to high effect sizes in both treatment groups and no differences in treatment satisfaction between the groups (Bruinsma et al., 2016).

Blended interventions appear increasingly popular as treatment protocols for blended therapy become more widely available (Kemmeren et al., 2016; Kleiboer et al., 2016; Kooistra et al., 2014; Nakao et al., 2018; Romijn et al., 2015). However, little is known about therapist fidelity to such protocols. Therapist fidelity is defined as the extent to which treatment is carried out as outlined in the treatment manual (Waltz et al., 1993). A lack of adequate evaluation of fidelity to blended treatment protocols can lead to incorrect conclusions about their clinical effectiveness (Prowse, 2015), because there is no way of knowing what exactly took place during the therapy. A further aim of bCBT is to improve cost-effectiveness through reduced therapist time, replacing a portion of the face-to-face sessions with online sessions. However, no clear indications for savings in terms of time or costs have been found up to now (Bruinsma et al., 2016; Kenter et al., 2015; Romijn et al., n.d). Whether or not bCBT has been applied as designed is a key question for cost-effectiveness analysis, as it has important implications for the interpretation of cost outcomes. Suboptimal bCBT implementation may actually lead to less effective ways of treating patients. For example, in a naturalistic study by Kenter and colleagues (Kenter et al., 2015), which evaluated the use of blended treatment for anxiety and depression in routine mental health care settings, treatment time and costs increased for bCBT relative to FtFCBT, because therapists delivered the online treatment on top of the FtF sessions.

The degree of treatment fidelity is important not only for understanding effectiveness and cost outcomes, but it also provides essential input for developing therapist guidelines on how to use bCBT (Bellg et al., 2004). In a recent qualitative study by Mol and colleagues (Mol et al., 2019), therapists (*N* = 36) pointed to a lack of clear guidelines on incorporating online sessions as a major barrier to providing bCBT.

Protocol use in FtFCBT for anxiety disorders has been evaluated, and results generally indicate high fidelity to treatment protocols (Boswell et al., 2013; Zickgraf et al., 2016). In these studies, audio-taped sessions (*N* = 495) (Boswell et al., 2013) or video-taped sessions (*N* = 39) (Zickgraf et al., 2016) were rated in terms of therapist adherence to the CBT protocol, with mean scores emerging of 85% on a 0%-to-100% scale (SD = 10.4) and 6.18 (SD = 0.51) on a 1-to-7 scale. Concerning Internet-based CBT, Hadjistavropoulos and colleagues investigated adherence to feedback instructions in online feedback messages (Hadjistavropoulos et al., 2018). They rated 706 messages for absence or presence of recommended therapist behaviours and found that adherence was generally high: seven out of nine behaviours were identified as present in 72–100% of messages. In a study by Mol and colleagues (Mol et al., 2018) therapist adherence to 219 written feedback instructions in online sessions within bCBT was investigated in patients with depressive disorder on a 0%-to-100% scale. They concluded that therapists adhered to most of the instructions relating to issues like structure (87.7%), readability (68%), writing style (93.6%) and communication skills (69.4%).

Although fidelity to FtFCBT and iCBT has been studied separately, no research on fidelity to bCBT has been conducted that takes both treatment modalities of the package into account. bCBT differs from FtFCBT and iCBT in that it requires therapists not only to apply therapeutic skills in both FtF and online sessions, but also to combine the two modalities into a single treatment. Several studies have shown that this integration of modalities can be challenging for therapists. In the naturalistic study by Kenter et al. (2015), for example, only a minority (18%) of therapists (*n* = 250) trained and equipped to use bCBT actually offered a treatment containing both FtFCBT and iCBT to their patients. Furthermore, they used iCBT as an add-on rather than a replacement of FtF sessions. In a qualitative study on barriers and facilitators of bCBT (Titzler et al., 2018) therapists (*n* = 5) stated that they lacked knowledge on how to integrate online components in FtF therapy and in a study on patient experiences with bCBT (*n* = 15), a deficiency in the interplay between FtF and online components and a lack of therapist awareness of patient activities in online sessions was found to be a cause of patient dissatisfaction (Urech et al., 2018). To improve the application of bCBT as intended, a key recommendation is to provide therapists with more guidelines on how to use bCBT (Mol et al., 2019).

The present study builds on the research reported by Romijn et al. (Romijn et al., n.d), in which therapists were provided with a bCBT protocol and clear guidelines on how to use bCBT. To explore whether this enables therapists to conduct a blended treatment as intended, we assess fidelity to (i) the blended format of the treatment (distribution and frequency of FtF and online sessions) and (ii) the instructions pertaining to the interplay between FtF and online sessions (such as the explanation of the format to patients, the assisted login to the online platform during the first FtF session, and the provision of CBT-specific feedback in response to each online session). In addition, we assess the relationship between blended treatment fidelity and specific patient characteristics which therapists reportedly perceive as making patients better suited for blended therapy: younger age, employment, computer skills, higher cognitive capacities, mild-to-moderate and less complex symptoms, and preference for bCBT over FtFCBT (Mol et al., 2019).

## 2 Methods

### 2.1 Design

Data were collected within an RCT assessing the clinical and cost-effectiveness of bCBT in comparison with FtFCBT (Romijn et al., 2015; Romijn et al., n.d). In that trial, 114 adult patients diagnosed with panic disorder, social anxiety disorder or generalised anxiety disorder were randomised to either bCBT (*n* = 52) or FtFCBT (*n* = 62) in one of four Dutch outpatient clinics for specialised mental health care between November 2015 and July 2017. Written informed consent was obtained from all participants before baseline assessment and randomisation. Participants were informed that participation in the trial was not contingent upon agreeing to be audio-recorded. A separate consent document for permission to record was obtained. A total of 45 patients started the bCBT treatment. Since one patient dropped out after the first session, our study of treatment fidelity analyses data from the 44 participants who actually received bCBT. The trial was approved by the Medical Ethics Committee of the Amsterdam University Medical Centers, location VU University Medical Center (registration number 2015.073) and registered in the Netherlands Trial Register (NTR4912).

### 2.2 Intervention

Separate manualised bCBT protocols were developed for patients with panic disorder, social anxiety disorder and generalised anxiety disorder (Romijn et al., 2015). Their content was based on protocols for FtFCBT (Keijsers et al., 2010), which contain evidence-based elements for the treatment of anxiety disorders, such as cognitive therapy and exposure (NICE 2011; NICE 2013) (see Appendix 1). In all blended treatments, both FtF and online sessions involved therapeutic guidance by qualified psychologists. The treatment consisted of 15 weekly sessions, with 8 FtF sessions alternating with 7 online sessions that were followed up by scheduled online feedback from the therapist. Every course of treatment began with a FtF session. Online sessions were accessible in a secure web-based environment (Minddistrict; www.minddistrict.com). Patients and therapists accessed this platform with a personalised login. The online sessions offered information (videos and text), testimonials from fictional patients, assignments (e.g., challenging negative thoughts) and homework exercises (e.g., monitoring activities, feelings, thoughts and behaviour). Therapists provided feedback on assignments and homework exercises. Default text templates for feedback and instructions were supplied for every online session as a therapist aid for providing feedback as intended. Therapists were free to tailore these texts to the specific needs of their clients. The online treatment platform also offered the option of repeating an online session. Therapists could decide on that if they deemed it beneficial, for example if the patient had not fully comprehended the content of an online session or had greatly benefited from a specific exercise in it.

Table 1 shows the protocol components for the FtF sessions and the online sessions. These contained instructions for the blended format, which were used to rate the extent of therapist fidelity.

### 2.3 Patients

Patients were invited for study participation if they (i) were aged 18 or older and (ii) met the DSM-IV criteria for panic disorder with or without agoraphobia, social anxiety disorder or generalised anxiety disorder, as diagnosed with the Structured Clinical Interview for DSM-IV Axis I Disorders (SCID-I (First et al., 2002)) or the Mini-International Neuropsychiatric Interview, Plus Version (MINI-Plus (Sheehan et al., 1998; Van Vliet and De Beurs, 2007)). Exclusion criteria were (i) inadequate proficiency in Dutch, (ii) lack of e-mail address or computer with Internet access and (iii) presence of a psychotic or bipolar disorder, substance dependence or a high risk for suicide.

### 2.4 Therapists

Therapists were trained and experienced in delivering CBT. Prior to treating patients in the trial, all therapists received a 2-h training course in the delivery of the blended treatment protocol, provided by researcher GR. During the training, therapists received instructions on how to apply the blended format, and were shown the main sections and functionalities of the online platform such as the start page of the therapist portal and entry point to other sections of the platform, treatment modules, homework area, feedback templates and messaging function. They also had the chance to practise with a fictitious patient. In addition, discussion sessions were organised by the research team in which therapists could exchange their experiences with bCBT.

### 2.5 Measures

Information on patient characteristics was obtained via an online self-report questionnaire at baseline. Primary diagnosis and comorbid disorders were established by a diagnostic interview (SCID-I (First et al., 2002) or MINI-Plus (Sheehan et al., 1998; Van Vliet and De Beurs, 2007)). Data on the dose and timing of FtF sessions were extracted from electronic medical records. Data on the dose and timing of online sessions were collected through the online treatment platform. FtF sessions were audio-recorded if participants consented, and these were transcribed verbatim. Feedback messages sent by therapists after online sessions were obtained from the online platform. All data were entered into Microsoft Office Excel (2016) spreadsheets.

### 2.6 Fidelity to ratio of blending

The ratio of blending for each course of treatment was calculated as the distribution of FtF and online sessions in percentages (ratio of blending = % FtF sessions / % online sessions).

### 2.7 Fidelity to session frequency

The bCBT protocol prescribed weekly sessions. Session frequency was determined by dividing the total number of completed FtF and online sessions by the total therapy duration in weeks (session frequency = (number of completed FtF sessions + number of completed online sessions) / duration therapy in weeks)).

### 2.8 Fidelity to blended protocol instructions

To assess fidelity to the blended protocol in the FtF sessions and the online sessions, we developed a checklist based on the treatment protocol, containing the mandatory blended protocol components (Table 1; see Appendix 2 for the complete checklist). Transcripts of treatment recordings and online written feedback messages were evaluated by two independent raters (GR, SP), who quantified the extent of therapist fidelity to the blended protocol instructions. One of the raters was a researcher involved in developing the treatment and conducting the trial, and one was an independent researcher not involved in the trial or in developing the treatment. Rating results were discussed until agreement was established. Interrater reliability was measured with intraclass correlation coefficients (ICC). The ICC for fidelity ratings of the FtF protocol components was 0.90 (*p* < .001, 95% CI 0.86–0.93) and for online components 0.90 (*p* < .001, 95% CI 0.88–0.91), indicating good agreement between the raters with respect to both FtF and online sessions.

### 2.9 Analysis

Descriptive statistics (means, standard deviations, percentages) were used to describe the sample and the observed fidelity. The relationships between fidelity scores and patient characteristics were assessed with Pearson correlations. Analyses were undertaken using SPSS, version 23 (IBM Inc., USA).

## 3 Results

### 3.1 Patient and therapist characteristics

A total of 44 patients were given bCBT by 28 therapists. Baseline demographics and clinical characteristics of the patient sample can be found in Table 2. Of the 28 therapists 24 were female (86%), most were licensed health care psychologists (54%, 15/28). Others were psychologists in training for health care psychologist and under supervision (46%, 13/28).

### 3.2 Blending ratio and session frequency

The mean treatment duration was 12.6 sessions (SD: 5.2), with 6.7 FtF sessions (SD: 2.6) and 6.0 online sessions (SD: 2.9) in 15.6 weeks (SD: 8.7). The mean percentage of FtF sessions in the 44 courses of blended treatment (55%) was almost equal to the prescribed 53%. Most courses of treatment (64%, *n* = 29) contained 50% to 60% FtF sessions. In fourteen cases (32%), the share of FtF sessions was either 40% to 50% (18%, *n* = 8) or 60% to 70% (14%, *n* = 6); in two cases (5%) it was higher than 70% (75% and 80%).

The mean session frequency was 0.89 sessions per week (SD: 0.26), slightly lower than the 1.0 sessions per week as prescribed. Five courses of treatment (11%) had a frequency of exactly 1 session per week, in 13 cases (30%) frequency was higher (range: 1.1–1.4) and in 26 cases (59%) frequency was lower (range: 0.3–0.9).

Session frequency was higher (0.98 sessions/week, SD: 0.17) when the ratio of online to FtF sessions was positive than when the ratio of FtF to online sessions was positive (0.85 sessions/week, SD: 0.28). The correlation between session frequency and the FtF-to-online ratio was significant (*r* = 0.373, *p* = .013), suggesting that patients who received a larger share of online sessions were likely to have higher-frequency treatment than those receiving more FtF sessions.

### 3.3 Fidelity to the blended protocol in FtF sessions

A total of 293 FtF sessions were conducted. Recordings were available for 74 (25%) sessions received by 23 patients. There were no significant differences in baseline characteristics (age, gender, education level, employment, primary diagnosis, baseline symptom scores, comorbidity, treatment preference and computer experience) between the patient sample that consented with recordings and the patient sample that did not consent with recordings. The recorded sessions were provided by 17 therapists, of whom 15 were female (88%), 10 (59%) were licensed health care psychologists and 7 (41%) were supervised psychologists in training for health care psychologists. Reasons for non-availability of recordings included: participants not consenting to be recorded (*n* = 21, 130 sessions), therapists forgetting to record sessions, poor recording quality and low battery of the recording device. Forty-six (62%) recorded sessions were conducted in full accordance with the blended instructions in the protocol, while in 28 sessions (38%) some devations to the protocol occurred (see Table 3 for adherence to the specific protocol components).

Blended protocol instructions for the psychoeducation component, which compared to the other two components only occurred in the first session, were adhered to in 7 of 8 recorded sessions (see Appendix 3, Box 1 for an example). All therapists assisted the patient in logging into the platform during the first session to introduce the online treatment programme, but in one session the therapist did not mention the alternation of FtF and online sessions in the blended treatment.

In 5 of 66 recorded sessions (7%), therapists deviated from the protocol where the previous online session should have been discussed (Boxes 2a and 2b). In one case the online session was not mentioned at all; in other cases therapists did refer to it, but there was little or no discussion of homework and assignments.

Most deviations from the protocol occurred when the upcoming online session was to be discussed (Box 3). In 22 of 66 sessions (33%), therapists deviated from the instructions, mostly by not scheduling an appointment for providing feedback on the next online session (30%, *n* = 20). In the other two cases, therapists did not discuss the homework for the upcoming online session.

### 3.4 Fidelity to the blended protocol in online sessions

Therapists provided a total of 257 feedback messages on 257 online sessions (see Table 4 for adherence to specific protocol components). In 167 messages (65%), blended protocol instructions were fully adhered to, meaning that the therapist had provided both generic therapeutic and CBT-specific feedback, and had scheduled the appointment for the next FtF session (see Appendix 3, Boxes 4a and 4b for examples). Generic therapeutic feedback was provided in 232 messages (90%), CBT-specific feedback in 184 messages (72%) and an appointment for the upcoming FtF session was scheduled in 208 messages (81%).

Thirty-four (13%) of the 257 online sessions were repeated. Feedback messages on repeated sessions were usually short and practical in nature (Boxes 5a to 5c) and usually did not contain CBT-specific feedback (31 of 34 messages).

### 3.5 Correlations of patient characteristics with treatment fidelity outcomes

Table 5 shows correlations between patient characteristics and fidelity outcomes. There was a significant association between the ratio of FtF to online sessions and a patient experience with the use of computers (*r* = −0.314, *p* = .038), indicating that the treatment of patients more experienced with computers was likely to contain a larger percentage of online sessions. No significant associations between other patient characteristics and fidelity outcomes were found.

## 4 Discussion

### 4.1 Principal findings

The aim of this paper was to explore therapist fidelity to bCBT protocols for anxiety disorders in specialised mental health care, considering insights in the actual application of blended treatment are lacking. Additionally, we wanted to gauge the influence of patient characteristics on bCBT fidelity, since therapists believe some patients are better suited for bCBT than others (Mol et al., 2019).

Overall, therapist adherence to the instructions in the blended treatment protocol was high. The mean session frequency was 0.89 sessions per week (SD: 0.26), slightly lower than the 1.0 sessions per week as prescribed. The ratio of FtF to online sessions was negatively associated with session frequency, suggesting that a larger share of online sessions enables a higher treatment frequency. This may be relevant in the light of meta-analytic findings by Cuijpers and colleagues (Cuijpers et al., 2013) showing the importance of treatment frequency: they found that an increase from one to two sessions per week in psychotherapy for depression boosted the effect size *g* by 0.45, with the total number of sessions held constant.

Our inspection of patient characteristics showed a significant association between the ratio of blending and patient experience with the use of computers, indicating that those with more computer experience were more likely to receive a higher share of online sessions. Other patient characteristics, such as pretreatment anxiety severity or comorbidity, were not associated with fidelity outcomes. This finding is in line with previous findings for FtF therapy (Boswell et al., 2013; Zickgraf et al., 2016) and refutes therapists' belief that patients with mild-to-moderate and less complex symptoms are better suited for bCBT (Mol et al., 2019).

### 4.2 Strengths and limitations

Evaluating fidelity is a time-consuming process, and it becomes even more complex when two treatment modalities are integrated into one treatment protocol. For this reason, treatment fidelity is often not examined in intervention studies, and had not yet been evaluated for blended treatment at all, even though it is essential to the interpretation of treatment outcomes and to successful implementation of a blended format. As this study was an investigation of therapist fidelity to a blended treatment protocol assessing both the FtF and the online elements of the treatment, we were able to examine what actually happened during blended treatment: did it blend?

It should be taken into account that the current analyses into fidelity were explorative in nature and should be seen as one of the first steps in unravelling the application of blended treatment. Although findings on blending ratio, session frequency and adherence to protocol instructions in online sessions were based on the full patient sample, results regarding the adherence to instructions in FtF sessions were based on a subsample of patients and a subset of therapy sessions. Even though characteristics of this subsample of patients resembled the full sample, we cannot be sure whether the recorded sessions were representative of all FtF sessions, which is a clear limitation of the study.

Furthermore, comparing our results to other studies, and comparing outcomes on treatment fidelity in general, is complicated by the lack of uniformity in the definition of fidelity used by different authors. One general definition is: ‘the degree to which a treatment is implemented as it was intended in the original protocol’; however, more specified definitions vary across studies and varying interpretations of the concept hinder shared understanding of findings (Gearing et al., 2011). The current study was the first to target fidelity assessment to the instructions aiming to achieve the blended format and interplay between FtF and online sessions. This limits generalizability to studies that take a broader view in defining fidelity and which were different in nature as they did not entail an Internet component as part of the treatment. However, the operationalisation of ‘fidelity’ applied in our study may be used as an indicator for other studies investigating fidelity in blended interventions and thus offers a helpful starting point to further unravel the black box of blended treatment.

### 4.3 Clinical and research implications

Opportunities to improve therapist fidelity to the blended treatment format appear to lie in enhancing therapist recognition of FtF and online sessions as equally important elements of treatment. If that is not acknowledged, online sessions cannot adequately replace FtF sessions. In the current study, therapists often did not set a date to provide feedback on online sessions, which could be an indication that a therapist sees those sessions as merely supportive to the FtF sessions and this idea can uncounsiously be transferred to the patient. This requires attention in training therapist. Furthermore, if an appointment calendar function were added to the online platform, that might improve fidelity to this protocol component and heighten therapists' awareness of the importance of online elements in blended treatment.

Previously, a lack of clear guidelines has been identified as a barrier to the use of bCBT (Mol et al., 2019; Titzler et al., 2018). In some cases in the current study, treatment frequency was higher than the intended one session per week, and that higher frequency was sometimes caused by a lack of clarity about how to integrate the online element into the treatment. This points to the need for clear instructions about online communication (such as how to deal with flexible, on-demand online contact opportunities and how much therapist time is available for online activities) and to the necessity of more intensive therapist training, which can prevent bCBT from becoming too demanding for therapists or too costly.

One benefit of a blended format, as found in earlier studies, is that it can enhance therapists' adherence to the treatment protocol (Mol et al., 2019; Titzler et al., 2018). In the current study, we indeed found high therapist fidelity in most cases. The overall variability in ratio of blending and session frequency, however, was quite high. This could be an indication that some therapists feel the need for a more flexible protocol to be able to adapt to patient preferences and needs. The character of online sessions facilitates flexibility in shortening or expanding (the online part of) treatment or vary in therapy frequency. Offering a customisable blended protocol has been suggested before (Kemmeren et al., 2019; Titzler et al., 2018; van der Vaart et al., 2014; Wentzel et al., 2016), and future research should further explore this option and investigate what degree of flexibility might be feasible.

Finally, an interesting topic for subsequent research would be whether therapist variables are associated with the degree of treatment fidelity. Identifying therapist characteristics that predict fidelity to bCBT could assist mental health care services in the selection and training of professionals.

## 5 Conclusions

Our findings suggest that the blended treatment was generally conducted as intended, indicating that delivery of bCBT in the applied format is feasible for therapists in specialised mental health care. This enhances confidence in the findings on effectiveness and cost-effectiveness of bCBT reported elsewhere (Romijn et al., 2015; Romijn et al., n.d): high treatment fidelity improves internal validity (participants in the experiment group actually received the treatment variable as intended) and external validity (the treatment can be replicated because the protocol was followed) (Borrelli, 2011). The results should, however, be interpreted with some level of caution, given that the findings on fidelity in FtF sessions were not based on the full patient sample.

The current study was conducted prior to the coronavirus crisis. The outbreak of a pandemic disease highlights the relevance of online treatment as an important element of routine care practice (Wind et al., 2020). Blended interventions are likely to be of critical importance in post-corona mental health care.

The following are the supplementary data related to this article.

Supplementary Table 1 Protocol components with instructions regarding the blended format. Supplementary Table 1

Supplementary Table 2 Patient characteristics. Supplementary Table 2

Supplementary Table 3 Adherence to protocol instructions in face-to-face sessions (*n* = 74 recorded sessions). Supplementary Table 3

Supplementary Table 4 Adherence to protocol instruction in online sessions (*n* = 257 sessions). Supplementary Table 4

Supplementary Table 5 Correlations of patient characteristics with treatment fidelity outcomes (*n* = 44, Pearson's *r*). Supplementary Table 5

Appendix 1 Content of the sessions in the bCBT protocol. Appendix 1

Appendix 2 Checklist for fidelity to bCBT treatment protocol, including examples. Appendix 2

Appendix 3 Examples of full and partial adherence to blended instructions in FtF sessions and online feedback messages Appendix 3

## Declaration of competing interest

The authors report no conflicts of interest related to this publication.
