# Supplementary material for: Does it blend? Exploring therapist fidelity in blended CBT for anxiety disorders
Source: Internet Interv. 2021 Jun 26;25:100418. doi: 10.1016/j.invent.2021.100418 (PMC8350592; doi:10.1016/j.invent.2021.100418)
Supplement: Supplementary Table 1 — Protocol components with instructions regarding the blended format. [file mmc1.docx]

| **Table 1. Protocol components with instructions regarding the blended format** | | |
| --- | --- | --- |
| **FtF sessions** | | |
| **Protocol component** | **Session** | **Instructions** |
| Psychoeducation: explanation of anxiety disorder, treatment and blended approach | 1 | - Provide explanation of treatment format: alternating FtF and online sessions - Log in to online platform together with patient to provide a technical introduction |
| Discussing previous online session | 3–15 | - Discuss homework and assignments from previous online session |
| Preparing upcoming online session | 1–13 | - Discuss homework and content of next online session - Schedule appointment for providing feedback on next online session |
| **Online sessions** | | |
| Generic therapeutic feedback | 2–14 | Provide feedback containing therapist behaviour that would be used in any psychotherapy intervention, such as   - encouraging and motivating - normalising - empathising - confirming by summarising |
| CBT-specific feedback | 2–14 | Provide CBT-driven feedback, such as   - helping patient identify and test automatic thoughts - helping patient identify and modify core beliefs - helping patient plan and conduct behavioural experiments |
| Scheduling upcoming FtF session | 2–12 | Schedule an appointment for next FtF session or remind patient of the already scheduled appointment |

_FtF: face-to-face_
