# Supplementary material for: Does it blend? Exploring therapist fidelity in blended CBT for anxiety disorders
Source: Internet Interv. 2021 Jun 26;25:100418. doi: 10.1016/j.invent.2021.100418 (PMC8350592; doi:10.1016/j.invent.2021.100418)
Supplement: Supplementary Table 2 — Patient characteristics. [file mmc2.docx]

**Table 2. Patient characteristics**

| **Patient characteristics (*N* = 44)** | |
| --- | --- |
| Age in years, mean (SD; range) | 36.7 (11.0; 19–62) |
| Gender female, *n* (%) | 23 (52) |
| Higher education*, *n* (%) | 12 (27) |
| Employment, *n* (%) | 28 (63.2) |
| Primary diagnosis, *n* (%)  Panic disorder  Social anxiety disorder  Generalised anxiety disorder | 23 (52.3)  11 (25.0)  10 (22.7) |
| BAI score at baseline, mean (SD) | 28.2 (11.6) |
| Comorbid disorder**, *n* (%) | 25 (57) |
| Preference for bCBT over FtFCBT, *n* (%) | 24 (55) |
| Weekly hours of computer use, mean (SD) | 20.5 (18.4) |
| _SD: standard deviation; BAI: Beck Anxiety Inventory (Beck et al., 1988; Fydrich et al., 1992) ; bCBT: blended cognitive-behavioural therapy; FtFCBT: face-to-face cognitive-behavioural therapy_  _*_ _Bachelor’s equivalent or higher_  _** Comorbid disorders: social phobia, panic disorder, agoraphobia, generalised anxiety disorder, major depressive disorder, dysthymia, posttraumatic stress disorder, obsessive-compulsive disorder, eating disorder_ | |
