# Supplementary material for: Does it blend? Exploring therapist fidelity in blended CBT for anxiety disorders
Source: Internet Interv. 2021 Jun 26;25:100418. doi: 10.1016/j.invent.2021.100418 (PMC8350592; doi:10.1016/j.invent.2021.100418)
Supplement: Supplementary Table 3 — Adherence to protocol instructions in face-to-face sessions (n = 74 recorded sessions). [file mmc3.docx]

**Table 3. Adherence to protocol instructions in face-to-face sessions (n=74 recorded sessions)**

| **Protocol component** | **Protocol instruction for blended approach** | **Adherence** | **Deviations from protocol** |
| --- | --- | --- | --- |
| Psychoeducation (*n* = 8) | Explain treatment format: alternating FtF* and online sessions, and login to online platform together with patient | Full adherence: 7  Partial adherence: 1  Non-adherence: 0 | - Therapist did not mention the alternating FtF and online sessions (1) |
| Discussing previous online session (*n* = 66) | Discuss homework and assignment(s) from previous online session | Full adherence: 61  Partial adherence: 4  Non-adherence: 1 | - Session was mentioned, but with little or no discussion of homework and assignments (4) - Previous online session was not mentioned at all (1) |
| Preparing upcoming online session (*n* = 66) | Discuss homework for next online session and schedule time for feedback provision | Full adherence: 44  Partial adherence: 21  Non-adherence: 1 | - No time for providing feedback was scheduled (20) - Homework for upcoming online session was not discussed (1) - Upcoming online session was not mentioned at all (1) |

^FtF: face-to-face^
