# Supplementary material for: Does it blend? Exploring therapist fidelity in blended CBT for anxiety disorders
Source: Internet Interv. 2021 Jun 26;25:100418. doi: 10.1016/j.invent.2021.100418 (PMC8350592; doi:10.1016/j.invent.2021.100418)
Supplement: Supplementary Table 4 — Adherence to protocol instruction in online sessions (n = 257 sessions). [file mmc4.docx]

**Table 4. Adherence to protocol instruction in online sessions (n=257 sessions)**

| **Protocol component** | **Online sessions with full adherence to instructions** |
| --- | --- |
| Generic therapeutic feedback | *n* = 232 (90%) |
| CBT-specific feedback | *n* = 184 (72%) |
| Scheduling upcoming FtF session | *n* = 208 (81%) |

^CBT: cognitive behavioural therapy; FtF: face-to-face^
