# Supplementary material for: Does it blend? Exploring therapist fidelity in blended CBT for anxiety disorders
Source: Internet Interv. 2021 Jun 26;25:100418. doi: 10.1016/j.invent.2021.100418 (PMC8350592; doi:10.1016/j.invent.2021.100418)
Supplement: Supplementary Table 5 — Correlations of patient characteristics with treatment fidelity outcomes (n = 44, Pearson's r). [file mmc5.docx]

**Table 5. Correlations of patient characteristics with treatment fidelity outcomes (n=44, Pearson’s *r*)**

| **Patient characteristics** | **Blending ratio** | **Session**  **frequency** | **FtF fidelity score (*n* = 23)** | **Online fidelity score** |
| --- | --- | --- | --- | --- |
| Age | .213 | .163 | −.083 | .244 |
| Higher education | −.104 | .159 | −.340 | −.111 |
| Employed | .016 | .130 | −.110 | −.157 |
| Baseline BAI score | .238 | −.290 | −.024 | .114 |
| Comorbid disorder | −.227 | −.023 | .080 | −.275 |
| Preference for bCBT | .161 | .092 | .228 | −.132 |
| Weekly computer hours | −.314^*^ | .140 | .024 | −.159 |

_BAI: Beck Anxiety Inventory; bCBT: blended cognitive-behavioural therapy; FtFCBT: face-to-face cognitive-behavioural therapy_

_*_ *_p_* _< .05_
