# Supplementary material for: Does it blend? Exploring therapist fidelity in blended CBT for anxiety disorders
Source: Internet Interv. 2021 Jun 26;25:100418. doi: 10.1016/j.invent.2021.100418 (PMC8350592; doi:10.1016/j.invent.2021.100418)
Supplement: Appendix 1 — Content of the sessions in the bCBT protocol. [file mmc6.docx]

**Appendix 1. Content of the Sessions in the bCBT Protocol**

| **Diagnosis** | ***Session***  ***(modality)*** | **Content and exercises** |
| --- | --- | --- |
| **Panic disorder** | *1 (FtF)*  *2 (online)*  *3 (FtF)*  *4 (online)*  *5 (FtF)*  *6 (online)*  *7 (FtF)*  *8 (online)*  *9 (FtF)*  *10 (online)*  *11 (FtF)*  *12 (online)*  *13 (FtF)*  *14 (online)*  *15 (FtF)* | Introduction, psychoeducation  Treatment rationale, treatment motivation and expectation, worry diary  Explanation of exposure, instructions regarding interoceptive exposure, panic diary  Interoceptive exposure exercises, exposure diary  Explanation of interoceptive exposure and exposure in vivo, exposure exercise, exposure diary  Identifying automatic thoughts, challenging unhelpful thoughts, exposure diary  Explanation of behavioural experiments, treatment evaluation, exposure diary  Identifying cognitive distortions, behavioural experiment, exposure diary  Behavioural experiment, exposure diary  Exposure exercises, behavioural experiments  Exposure exercises, behavioural experiments  Exposure exercises, behavioural experiments  Exposure exercises, behavioural experiments  Explanation of relapse prevention, relapse prevention plan  Relapse prevention plan, recapitulation, treatment evaluation |
| **Social anxiety disorder** | *1 (FtF)*  *2 (online)*  *3 (FtF)*  *4 (online)*  *5 (FtF)*  *6 (online)*  *7 (FtF)*  *8 (online)*  *9 (FtF)*  *10 (online)*  *11 (FtF)*  *12 (online)*  *13 (FtF)*  *14 (online)*  *15 (FtF)* | Introduction, psychoeducation  Treatment rationale, treatment motivation and expectation, worry diary  Explanation of selective attention, explanation and instructions regarding exposure, social anxiety diary  Identifying automatic thoughts, challenging unhelpful thoughts, social anxiety diary  Identifying automatic thoughts, challenging unhelpful thoughts, social anxiety diary  Identifying cognitive distortions, social anxiety diary  Explanation of behavioural experiments, treatment evaluation  Behavioural experiments, social anxiety diary  Behavioural experiments, social anxiety diary  Behavioural experiments, social anxiety diary  Behavioural experiments, social anxiety diary  Behavioural experiments, social anxiety diary  Behavioural experiments, social anxiety diary  Explanation of relapse prevention, relapse prevention plan  Relapse prevention plan, recapitulation, treatment evaluation |
| **Generalised anxiety disorder** | *1 (FtF)*  *2 (online)*  *3 (FtF)*  *4 (online)*  *5 (FtF)*  *6 (online)*  *7 (FtF)*  *8 (online)*  *9 (FtF)*  *10 (online)*  *11 (FtF)*  *12 (online)*  *13 (FtF)*  *14 (online)*  *15 (FtF)* | Introduction, psychoeducation  Treatment rationale, treatment motivation and expectation, worry diary  Explanation and instructions regarding exposure, challenging unhelpful thoughts, explanation of metacognitions  Exploring metacognitions, worry exposure exercises  Exploring uncontrollability of worrying  Worry experiment regarding uncontrollability  Exploring the danger of worrying, treatment evaluation  Worry experiment regarding danger of worrying  Exploring positive beliefs about worrying  Worry experiment regarding positive beliefs  Explanation of selective attention  Learning to shift attention  Learning to shift attention  Explanation of relapse prevention, relapse prevention plan  Relapse prevention plan, recapitulation, treatment evaluation |
