# Supplementary material for: Does it blend? Exploring therapist fidelity in blended CBT for anxiety disorders
Source: Internet Interv. 2021 Jun 26;25:100418. doi: 10.1016/j.invent.2021.100418 (PMC8350592; doi:10.1016/j.invent.2021.100418)
Supplement: Appendix 2 — Checklist for fidelity to bCBT treatment protocol, including examples. [file mmc7.docx]

**Appendix 2: Checklist for Fidelity to bCBT Treatment Protocol, Including Examples**

For FtF protocol components, a score of 2 indicates full adherence, 1 partial adherence and 0 non-adherence. For online protocol components, a score of 1 indicates presence of the component and 0 indicates non-presence.

| **FtF sessions** | | | |
| --- | --- | --- | --- |
| **Protocol component** | **Checklist**   - full adherence: score 2 - partial adherence: score 1 - non-adherence: score 0 | **Examples from transcripts** | |
| **Psychoeducation** | Adherence: therapist gives a clear explanation of the blended format and logs into the online platform with the patient | Session 1 (panic disorder, PD)  *Therapist: This treatment will last 15 weeks and FtF sessions will alternate with online sessions.*  *...*  *Therapist: Let’s take a look at the online platform.*  *Patient: Okay.*  *Therapist: It’s really easy, so you’ll be fine. Here you see the sessions and your progress, so you know where to start when you log in again. Now we’ll have a look at the introduction session together.*  *Patient: Yes, I can see that in Tasks.*  *Therapist: That’s right, you can see the introduction sessions there. And here you see what the content of the session is.*  *Patient: I see.* | |
|  | Partial adherence: therapist does not give a clear explanation of the blended format *or* does not log into the online platform with patient |  | |
|  | Non-adherence: therapist does not give a clear explanation of the blended format *and* does not log into the online platform with patient |  | |
| **Discussing previous online session** | Adherence: content of homework and exercises in the previous online session is discussed | Session 5 (PD)  *Therapist: I gave you some feedback on the online session. You had made a list of exposure activities and described your catastrophic thought. You described what happens when you get into your car very clearly. You think: “Oh no, I will stop at the next petrol station.”*  *Patient: That’s right.*  *Therapist: I think those are reactions to your catastrophic thought. It’s not that thought that makes you feel anxious and that makes you want to stop driving the car. Because ... what might happen if you keep driving? What’s the most catastrophic thing that could happen?* | |
|  | Partial adherence: reference is made to the online sessions, but content of homework and exercises is not discussed | Session 3 (PD)  *Therapist: First I want to briefly discuss the online session. How did that go?*  *Patient: I recognised some things, some of these exercises I’ve done before. And um ... I found it difficult to describe my own situation.*  *Therapist: Yes.*  *Patient: That’s still difficult for me. The rest of it was clear. When you start working on it, you start thinking about your situation. That’s good, I think....*  *Therapist: I read it and gave you feedback. How did you feel about that?*  *Patient: I found it supportive.*  *Therapist:Good.* | |
|  | Non-adherence: homework and exercises in the previous online sessions are not discussed |  | |
| **Preparing upcoming online session** | Adherence: homework for the upcoming online session is discussed and an appointment for providing feedback is made | Session 3 (PD)  *Therapist: This week you’ll keep your panic diary; you can do that online. That means you describe every panic attack in your diary. For example, if you have trouble breathing again and that makes you feel anxious.*  *...*  *Therapist: I’ll send you feedback on the online session next Monday, in the morning. Is that okay?*  *Patient: Yes.* | |
|  | Partial adherence: homework for the upcoming online session is not discussed *or* no appointment for providing feedback on the online sessions is made | Session 5 (PD)  *Therapist: We’ll see each other again on Wednesday in two weeks’ time.*  *Patient: Yes, that means I can do the exposure activity at least five times.*  *Therapist: That’s good, you can practise a lot.*  🡪 An appointment for the next FtF session is made, but not one for providing feedback on the online session. | |
|  | Non-adherence: homework for the upcoming online sessions is not discussed *and* no appointment for providing feedback on the online sessions is made |  | |
| **Online sessions** | | |  |
| **Protocol component** | **Checklist**   - presence of protocol component: score 1 - non-presence of component: score 0 | **Examples from feedback messages** |  |
| **Generic therapeutic feedback** | Encouraging and motivating | - *I see that you worked very hard!* - *Good job!* - *Well done!* |  |
|  | Normalising and empathising | - *I can imagine that made you sad and anxious.* - *I understand that it feels like a disappointment.* - *I can imagine that is not easy.* - *Simply put, people feel anxiety in their bodies sometimes, and especially people who are recovering from an anxiety disorder will regularly feel restless and anxious. That can’t be prevented.* - *Be aware that you don’t have to understand and be able to perform things perfectly at once. I’ve never met anyone who could (myself included), so it’s completely fine if you want to reassure yourself or need more time.* - *The goal was not (yet) to sit on your bike without fear (or fearful sensations). Because indeed, as you write, it takes time to get used to it, also for your body and your mind.* |  |
|  | Confirming by summarising | - *The most important part of the treatment is indeed to investigate your thoughts.* |  |
|  | Guiding treatment progress | - *You can log your panic attacks in the panic diary.* - *This week’s homework is that you will perform three activities from your exposure list.* - *Once you’ve completed an exercise, it’s good to log that in the exposure diary.* |  |
| **CBT-specific feedback** | Explaining CBT theory (including exposure) | - *Often you start by feeling something in your body: your heart starts beating faster, you start hyperventilating, and then you start feeling light-headed. Because you feel light-headed, you start thinking something bad could happen, for example fainting or having a heart attack. Then the reasoning becomes: If my heart beats faster, I will start hyperventilating, I’ll become light-headed, and then I’ll faint or have a heart attack.* - *The fear of fear became stronger, which made you end up in the panic cycle: anxiety 🡪 catastrophic thoughts about anxiety 🡪 anxiety increases 🡪 catastrophic thoughts become stronger 🡪 anxiety increases etc.* - *With an alternative thought we mean a thought that is more realistic than a catastrophic thought – keeping calming thoughts in mind. You can use those as well, but then they are more like helpful thoughts.* - *A more realistic thought is a combination of your catastrophic thought and common sense: If I start feeling dizzy, and start having palpitations, that means I’m starting to have a hyperventilation or panic attack, and not that I’ll get into an accident. Even though I think I’m losing control, this doesn’t mean it will actually happen (the chance of me having or causing an accident is smaller than I think).* |  |
|  | Guiding patients through CBT assignments (including exposure), for example by asking questions or providing information or suggestions | - *Is it correct that the believability of the catastrophic thought becomes weaker once the experiment has ended? So do you believe less strongly that you are becoming unwell?* - *I think that, based on what you told me earlier, perhaps we could think of other exercises. It’s not so much about all the things you’re afraid of doing, but about things you do differently because of your fear. That could be always making sure there is someone nearby that could help you. You can see that in the way you formulate your answers to the exercises: it is especially difficult to do things on your own.* - *The moment that you flee after all, or start measuring before the tension has gone down – that is no longer exposure, and it won’t work either. Moreover, the anxiety will just increase. One solution, I think, is to think of achievable steps and of using things that can aid you, in such a way that you can follow through.* - *In the evaluation of your experiment you write that you tend to overestimate negative outcomes, so that situations can be scary beforehand. What can you do to reduce this fear? What exercises from the treatment can you use to do this?* - *Of course, it could happen that someone does get angry when you don’t know something or say something wrong. How would you feel about that? Could you handle it? What could help you with that?* |  |
| **Scheduling appointment for FtF session** |  | - *We’ll see each other on Thursday 26th November at 13:00, see you then!* |  |
|  | | **Examples from feedback messages non-adherent to instructions** |  |
|  | | - *We already discussed this session during our FtF session.* - *I will open the next session for you.* - *We will discuss the assignments when we see each other again.* |  |
