# Supplementary material for: Does it blend? Exploring therapist fidelity in blended CBT for anxiety disorders
Source: Internet Interv. 2021 Jun 26;25:100418. doi: 10.1016/j.invent.2021.100418 (PMC8350592; doi:10.1016/j.invent.2021.100418)
Supplement: Appendix 3 — Examples of full and partial adherence to blended instructions in FtF sessions and online feedback messages [file mmc8.docx]

**Appendix 3: Examples of Full and Partial Adherence to Blended Instructions in FtF Sessions and Online Feedback Messages**

Box 1. Example of adherence to blended instructions in FtF session, protocol component **Psychoeducation**

*Therapist: This treatment will last 15 weeks and face-to-face sessions will alternate with online sessions.*

*(…)*

*Therapist: Let’s take a look at the online platform.*

*Patient: Okay.*

*Therapist: It’s really easy, so you’ll be fine. Here you see the sessions and your progress, so you know where to start when you log on again. Now we’ll have a look at the introduction session together.*

*Patient: Yes, I can see that in Tasks.*

*Therapist: That’s right, you can see the introduction sessions there. And here you see what the content of the session is.*

*Patient: I see.*

Box 2a. Example of adherence to blended instructions in FtF session, protocol component **Discussing Previous Online Session**

*Therapist: I gave you some feedback in the online session. You had made a list of exposure activities and described your catastrophic thought. You described what happens when you get into your car very clearly. You think: “Oh no, I will stop at the next petrol station.”*

*Patient: That’s right.*

*Therapist: I think those are reactions to your catastrophic thought. It’s not that thought that makes you feel anxious and that makes you want to stop driving the car. Because ... what might happen if you keep on driving? What’s the most catastrophic thing that could happen?*

Box 2b. Example of partial adherence to blended instructions in FtF session, protocol component **Discussing Previous Online Session**

*Therapist: First I want to briefly discuss the online session. How did that go?*

*Patient: I recognised some things, some of these exercises I’ve done before. And um... I found it difficult to describe my own situation.*

*Therapist: Yes.*

*Patient: That’s still difficult for me. The rest of it was clear. When you start working on it, you start thinking about your situation. That’s good, I think....*

*Therapist: I read it and I gave you feedback. How did you feel about that?*

*Patient: I found it supportive.*

*Therapist: Good.*

Box 3. Example of adherence to blended instructions in FtF session, protocol component **Preparing Upcoming Online Session**

*Therapist: This week you’ll keep your panic diary, you can do that online. That means you describe every panic attack in your diary. For example, if you have trouble breathing again and that makes you feel anxious.*

*…*

*Therapist: I’ll send you feedback on the online session next Monday in the morning. Is that okay?*

*Patient: Yes.*

Box 4a. Example of feedback message in online session adhering to blended instructions

| **Feedback message** | **Protocol component** |
| --- | --- |
| *Your answers in the exercises are very clear, nice!*  *In the first exercise you described the panic attack clearly, and you notice that it helps to formulate alternative thoughts such as: Thousands of people take the bus, I used to do that as well, and that is normal behaviour. What you’re telling yourself is that it is not a dangerous situation, and that the sensations of fear are essentially unnecessary. Henceforth you’ll see that the catastrophic thought “I am fainting” goes down from a believability of 80% to 25%, and that the alternative thought becomes more believable, and that because of this you will feel less anxious.*  *Very good that you practised the “head shaking”. Just as you indicate at the end: this is an uncomfortable feeling. But the question is whether that also means that you are going to faint/die. You notice that the catastrophic thought goes down from a believability of 85% to 20%. It is not necessary to be anxious about those sensations.*  *Concerning the exposure list, it may be useful to look at an activity that you could perform daily. That would make it easier to practise (or more often) and to lower the threshold. That is important, because for the next FtF session you will perform three activities from your exposure list. Don’t forget to keep track of those activities in your exposure diary.*  *We’ll discuss how that went on Monday next week. Good luck and see you then!* | Generic therapeutic feedback  CBT-specific feedback  Generic therapeutic feedback + CBT-specific feedback  Scheduling appointment for FtF session |

Box 4b. Example of feedback message in online session adhering to blended instructions

| **Feedback message** | **Protocol component** |
| --- | --- |
| *Thank you for doing the exercises. Things are looking good! I will once again give you some tips for each exercise:*  *(4.1 Describe a panic attack). I see that you’re getting better at describing that. Under the heading Thoughts, I’d like to know whether you have any more thoughts that arise. To bring automatic thoughts to the surface you can try to replay the event like a short movie and to ask yourself: what am I thinking now? Because what? And then?*  *About the catastrophic thought: does that thought describe the essence of your feelings? I ask this because you indicate that you are scared, angry and sad. What is the essence of your anxiety? For example: the thoughts never go away, and then....*  *For the alternative thought: nice, and a positive description! What effect does that thought have on you? Would the feeling or behaviour be different to when you think the catastrophic thought?*  *(4.2 Interoceptive exposure) Well done, you already did 2 exercises! I also see that the believability of the catastrophic thought was strongly reduced after the exercise. So it’s not necessary to be afraid of physical sensations.*  *(4.3 Exposure list) Great that you already managed to fill in 10 activities. It’s important that the activities give you the opportunity to investigate the catastrophic thought, for example, “The thoughts never end”. If an activity is accompanied by a different catastrophic thought, then it’s good to describe it as well (if possible in the diary).*  *It’s important to hold on to the alternative thought, and how you would react and feel, in the back of your mind. That way you can really challenge the thoughts. Example: Hyperventilating 1 minute –*  *Catastrophic thought: I’m going to faint. Feeling: fear. Behaviour: sitting down and breathing calmly.*  *Alternative thought: I have all these physical sensations, but do not faint. Feeling: fear. Behaviour: continuing and enduring the hyperventilation*  *For the next face-to-face session, you will perform three activities from your exposure list. Don’t forget to keep track of those activities in your exposure diary.*    *We will discuss how things went on the 18th of May. Good luck and see you then!* | Generic therapeutic feedback  CBT-specific feedback  CBT-specific feedback  CBT-specific feedback  Generic therapeutic feedback  Scheduling appointment for FTF session |

Box 5a. Example of feedback message on a repeated session

*I gave you feedback on the online session during the last FtF session. You can now proceed to the next online session.*

Box 5b. Example of feedback message on a repeated session

*We completed this session together, because you needed some help with it.*

Box 5c. Example of feedback message on a repeated session

*Thanks for completing this session, you can now proceed to the next session.*
